# Human Behavior Analysis by Means of Multimodal Context Mining

**DOI:** 10.3390/s16081264

**Published:** 2016-08-10

**Authors:** Oresti Banos, Claudia Villalonga, Jaehun Bang, Taeho Hur, Donguk Kang, Sangbeom Park, Thien Huynh-The, Vui Le-Ba, Muhammad Bilal Amin, Muhammad Asif Razzaq, Wahajat Ali Khan, Choong Seon Hong, Sungyoung Lee

**Affiliations:** 1 Department of Computer Engineering, Kyung Hee University, Yongin-si 446-701, Korea; oresti@khu.ac.kr or o.banoslegran@utwente.nl (O.B.); cvillalonga@khu.ac.kr or cvillalonga@correo.ugr.es (C.V.); jhb@oslab.khu.ac.kr (J.B.); hth@oslab.khu.ac.kr (T.H.); dwkang@oslab.khu.ac.kr (D.K.); sbp@oslab.khu.ac.kr (S.P.); thienht@oslab.khu.ac.kr (T.H.-T.); lebavui@oslab.khu.ac.kr (V.L.-B.); mbilalamin@oslab.khu.ac.kr (M.B.A.); asif.razzaq@oslab.khu.ac.kr (M.A.R.); wajahat.alikhan@oslab.khu.ac.kr (W.A.K.); sylee@oslab.khu.ac.kr (S.L.); 2 Telemedicine Group, Center for Telematics and Information Technology, University of Twente, Enschede 7500AE, The Netherlands; 3 Department of Computer Architecture and Computer Technology, Research Center on Information and Communications Technology, University of Granada, Granada E18071, Spain

**Keywords:** human behaviour, context awareness, activity recognition, location tracking, emotion identification, machine learning, ontologies

## Abstract

There is sufficient evidence proving the impact that negative lifestyle choices have on people’s health and wellness. Changing unhealthy behaviours requires raising people’s self-awareness and also providing healthcare experts with a thorough and continuous description of the user’s conduct. Several monitoring techniques have been proposed in the past to track users’ behaviour; however, these approaches are either subjective and prone to misreporting, such as questionnaires, or only focus on a specific component of context, such as activity counters. This work presents an innovative multimodal context mining framework to inspect and infer human behaviour in a more holistic fashion. The proposed approach extends beyond the state-of-the-art, since it not only explores a sole type of context, but also combines diverse levels of context in an integral manner. Namely, low-level contexts, including activities, emotions and locations, are identified from heterogeneous sensory data through machine learning techniques. Low-level contexts are combined using ontological mechanisms to derive a more abstract representation of the user’s context, here referred to as high-level context. An initial implementation of the proposed framework supporting real-time context identification is also presented. The developed system is evaluated for various realistic scenarios making use of a novel multimodal context open dataset and data on-the-go, demonstrating prominent context-aware capabilities at both low and high levels.

## 1. Background

The last World Health Organization (WHO) global status report on noncommunicable diseases reveals that illnesses associated with lifestyle choices are currently the leading cause of death worldwide [1]. As a matter of fact, non-communicable diseases are responsible for more than two-thirds of the world’s deaths, with more than 40% of them representing premature deaths under the age of 70 years. Recognizing this seriously worrying epidemic scenario, the WHO has defined a clear roadmap to alter the course of the so-called “slow-moving public health disaster”. Most of the policies presented in this roadmap seek to change unhealthy lifestyles and harmful conduct, such as tobacco and alcohol use, insufficient physical activity or excessive salt intake, among others, through the application of sustained prevention and control mechanisms.

Standard behaviour change strategies require an active involvement of users for their self-monitoring. Despite self-monitoring mechanisms being well-founded from a theoretical point of view, many of them have been proven ineffective in practice [2]. The main reason for this inefficiency is the low level of planning, motivation and vigilance shown by regular patients and users of these self-tracking mechanisms. People regularly manifest discomfort while measuring, interpreting and annotating data, which eventually leads to a lack of interest in the report task [3]. Accordingly, more motivational, volitional and encouraging means are needed to ensure the adherence and commitment of users, while guaranteeing a reasonable level of accuracy in the monitoring process.

Modern technologies are called upon to play a key role in supporting advanced self-monitoring mechanisms for behaviour change [4]. In fact, the use of information and communication technologies has increasingly been fostered during recent years to facilitate the automatic and seamless monitoring of people’s behaviour. Diverse commonplace technologies, such as accelerometers or GPS sensors, have been proposed in a variety of commercial products to assess, for example, the number of steps the user took and when they have the most trouble sleeping [5,6,7]. These monitoring technologies normally consist of two parts: (1) electronic sensor devices capable of measuring and translating human physical and physiological responses into digital data; and (2) digital processing systems in charge of the gathering, storage and analysis of the data. Although there exist multiple solutions encapsulating both parts into a single device, e.g., a smartwatch for the detection of activity levels [8], it is also frequent to find them separately, e.g., an instrumented wearable bracelet for registering electrodermal activity and an app on a smartphone for processing these data and deriving the user’s stress level [9]. The most prominent monitoring systems have been provided at the research level, normally prototypes oriented to keep track of health and wellness states. Examples of these systems are [10] for detecting cardiovascular illnesses, [11] for alerting about physical conditions or [12] for tracking changes in the physiological responses of patients with chronic diseases.

Despite the rapid growth of digital monitoring technologies for behaviour monitoring, the vast majority of existing solutions are domain specific, and consequently, they have a relatively narrow application scope. This presents important technical and practical limitations, such as redundancy while deriving similar user-centric information, duplicity in the use of computational resources or difficulty in sharing and re-using data for the estimation of diverse contextual information. Moreover, many applications misleadingly claim to infer human behaviour in a broad sense when they merely detect some activity-related parameters, such as step counts, calories burned or floors climbed. From a behavioural science perspective, the behaviour of an organism can be defined as everything it does, including covert actions like thinking and feeling [13]. This makes the identification of human behaviour a rather complex task that certainly demands the analysis of multiple factors, including not only physical, but also emotional and social aspects. In light of these limitations, some attempts have recently been made towards a more comprehensive and multifaceted monitoring of human behaviour. For example, [14] introduces a middleware that allows for the recognition of activities and indoor mobility to support context-aware services. A context-driven approach was developed in [15] for profiling the user’s activity at multiple levels of granularity. The system combines motion reconstruction, location detection and activity identification to describe the user’s context. In [16], the authors present a platform to gather users’ psychological, physiological and activity information for mental health purposes. Some efforts are also being put towards the creation of commercial frameworks capable of digesting different types of behaviour-related contextual information, such as HealthKit [17], GoogleFit [18] or SAMI [19], yet most of these initiatives rely on third party applications and systems for inferring the behaviour information.

This work describes the multimodal context mining framework, an innovative means to support automatic behaviour tracking in a holistic manner through the continuous and comprehensive inference of multimodal human context from heterogeneous sensory data. A unique characteristic of the proposed framework consists of the two levels of context granularity it provides, namely low- and high-level contexts. Low-level context categories, such as user’s physical activity, emotional states and locations, are discovered by means of signal processing and machine learning mechanisms. These categories are combined in a more abstract level through ontological mechanisms in order to determine high-level representations of the user’s context. This paper further extends prior work [20,21,22] while providing an exhaustive evaluation of the potential and validity of the proposed solution. In this sense, this paper presents a more functional and applied perspective of the low-high level context dualism, as well as the mechanisms to obtain one out of the other. Similarly, it goes beyond prior contributions, which mainly elaborated on accelerometer data to infer the user’s physical activity in order to describe their behaviour. Conversely, this work reveals a more comprehensive picture of the user’s context by not only inferring physical, but also emotional and location information by using new sensing modalities, such as audio, video and geopositioning data. Furthermore, this work makes use of a more opportunistic sensing approach [23], based on a multimodal sensor setup, including technologies intended for not only outdoor, but also indoor scenarios. The rest of the paper is organized as follows. Section 2 describes the multimodal context mining framework and its main components. Section 3 presents a realization of the proposed framework, including the technologies used for its implementation. The performance of each context recognizer, as well the awareness capabilities of the platform as a whole are evaluated and discussed in Section 4. The main conclusions of this work are summarized in Section 5.

## 2. Multimodal Context Mining Framework

Current domain-specific solutions are seen to be certainly insufficient to deal with the magnitude of the behaviour analysis problem, thus making it necessary to rather use more holistic approaches to infer and analyse people’s conduct. In this context, it is devised Mining Minds [22], a novel digital health and wellness platform designed to seamlessly investigate and support people’s lifestyles by intelligently mining human’s daily living data generated through heterogeneous resources. The multimodal context mining framework presented in this paper plays a core role in Mining Minds for the transformation of heterogeneous sensory data into interpretable and actionable information from which behavioural patterns can be derived. Although this framework has originally been devised to operate in conjunction with other layers of the Mining Minds platform stack, it can nevertheless operate as an independent and decoupled engine for the inference and modelling of people’s context.

**Figure 1:**
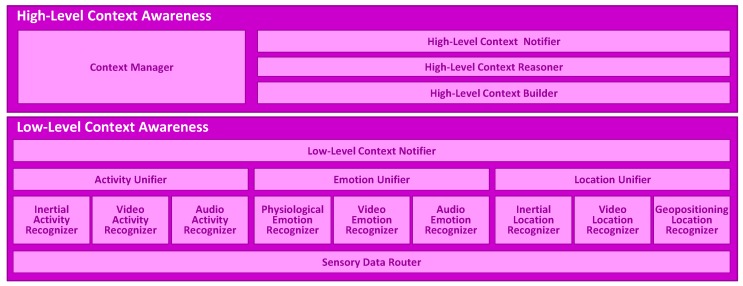
Architecture of the multimodal context mining framework.

The multimodal context mining framework is composed of two main modules (Figure 1), namely Low Level Context Awareness (LLCA) and High Level Context Awareness (HLCA). LLCA is in charge of converting the wide-spectrum of data obtained from the user interaction with the real and cyber world into abstract concepts or categories, namely physical activities, emotional states and locations. These categories are intelligently combined and processed at HLCA in order to determine and track more meaningful semantic representations of the user context. In the following, both the LLCA and HLCA architectures are thoroughly described.

### 2.1. Low-Level Context Awareness

**Figure 2:**
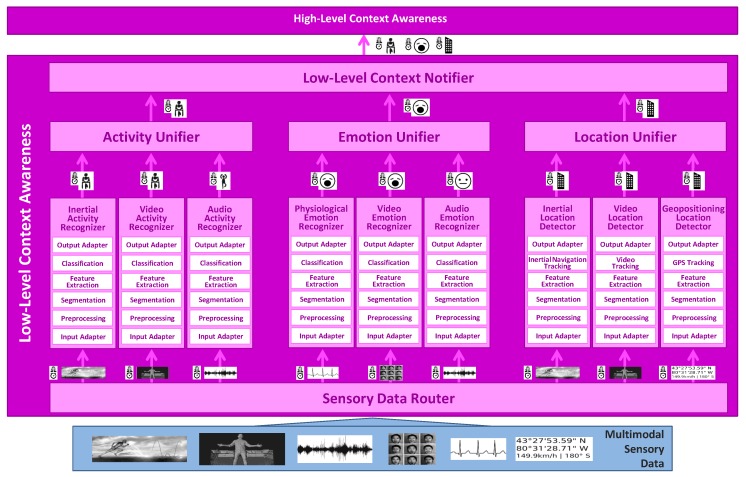
Low-Level Context Awareness operation flow.

LLCA consists of the Sensory Data Router, Low-Level Context Recognizers (i.e., Activity Recognizer, Emotion Recognizer, Location Recognizer), Low-Level Context Unifiers (i.e., Activity Unifier, Emotion Unifier, Location Unifier) and Low-Level Context Notifiercomponents. The operation of the LLCA module is as follows (Figure 2). The influx of sensory data coming from the monitoring devices is first processed by the Sensory Data Router component to identify the Low-Level Context Recognizers to which each datum must be distributed. This identification is performed based on the nature of the incoming data and the requirements of each recognizer. Upon receiving new data, the Activity Recognizer, Emotion Recognizer and/or Location Recognizer take action. Each of these recognizers includes various subcomponents operating on different sensor data modalities. The decisions delivered by each individual subcomponent are fused or unified into a single context for every category through the Activity Unifier, Emotion Unifier and Location Unifier, respectively. Finally, once a new low-level context is identified (either activity, emotion or location), the Low-Level Context Notifier makes it available to HLCA for further analysis and also to any potential third party application, which might be registered for this type of information.

#### 2.1.1. Sensory Data Router

The Sensory Data Router receives packets of sensory data consisting of raw data (samples) and metadata (data modality or type, time stamp and user ID). These packets are constructed in Mining Minds by the so-called Data Curation Layer [24], but can be equally supplied by any other entity (e.g., a wearable device or mobile application). A subscription mechanism is used for the distribution of the data. The Sensory Data Router uses a simple registry to cross-match the data type of a given sensory data packet with the specifications of each subscribed recognizer. This mechanism is found to be particularly appropriate for the broadcasting of data since multiple recognizers may potentially build on the same source (or combination) of data. The Sensory Data Router is not only responsible for feeding each recognizer with the appropriate data, but also dealing with the amount and frequency with which the data must be served. Regular buffers are used to temporarily cache the incoming data until the minimum amount of data required by a given recognizer to operate is reached or the recognizer appears to be ready for the processing of a new instance of data.

#### 2.1.2. Low-Level Context Recognizers

The transformation of the raw sensory data into low-level contexts, i.e., activities, emotions and locations, is carried out by the Low-Level Context Recognizers. The identification of the user physical activities is performed through the Activity Recognizer. Three subcomponents are actually in charge of processing and categorizing the input sensory data into elementary activity categories, such as “standing”, “walking” or “cycling”. These subcomponents are the Inertial Activity Recognizer, Video Activity Recognizer and Audio Activity Recognizer, whose names stem from the sensing modality in which each recognizer is specialized. Using multiple recognizers is intended here to support more flexible and opportunistic data collection settings, as well as to increase the accuracy and robustness in the activity identification.

Conceptually, the operation flow can be considered nearly similar for the three recognizers. Once a sensory data packet is received, the raw data or signals are separated from the metadata for further processing. Then, each signal is partitioned into smaller segments of data. Descriptors or features are extracted from each data segment to reduce their dimensionality and facilitate the subsequent classification or categorization process. Probabilistic models are used after training for the classification of the features into a specific activity kind or label. Finally, the original metadata accompanying the processed raw data are attached to the label to clearly identify the time and user linked to the recognized activity.

The Emotion Recognizer is designed to infer user emotional states, such as “happiness” or “disgust”, by using video and audio data, as well as human physiological data. As a result, three main subcomponents can be also identified here, namely Physiological Emotion Recognizer, Video Emotion Recognizer and Audio Emotion Recognizer. The operation flow for the various emotion recognizers is identical in architectural terms to the one described before for the activity recognizers.

Finally, the user situation is determined by the Location Recognizer, which builds on the data collected through indoor and outdoor positioning sensors, such as video, inertial and GPS sensors, to specify the exact location of the user. A given place may have a different meaning or description depending on the user (e.g., the home of a given person can be the office for another one). Therefore, rather than giving a generalized description of the user’s physical location, e.g., address, the Location Recognizer is intended to provide a more user-centric definition. To that end, user-centric points of interest are identified by using personalized maps. These maps are created by requesting users to provide the location of personal relevant places, such as their “home” or “office”. Then, a straight-forward mapping between physical and personalized locations is generally performed.

#### 2.1.3. Low-Level Context Unifiers

The individual decisions delivered by each recognizer are combined by the Activity Unifier, Emotion Unifier and Location Unifier to yield a single activity, emotion and location label, respectively. Due to the fact that each recognizer may generate decisions at different rates, e.g., the Inertial Activity Recognizer might produce a new activity label every couple of seconds whilst the Video Activity Recognizer might do so every half a minute, it is necessary to perform a vertical and horizontal fusion of decisions. The vertical fusion consists of individually aggregating all of the decisions delivered by each recognizer within a specific period of time. As a result, a single label is determined for each recognizer. Then, the horizontal fusion is performed for all of the recognizers of the same low-level context type, thus leading to an eventual decision or label representing the activity, emotion and/or location that have taken place during that specific time slot.

#### 2.1.4. Low-Level Context Notifier

The eventually recognized low-level contexts after unification are communicated to HLCA and also to potential third parties through the Low-Level Context Notifier. Notifications are only made effective whenever the newly-identified low-level context differs from the previous one for a specific category of context. Thus, for example, if the last activity performed by the user was “standing” and the one resulting from the unification is still “standing”, no notification would be generated. This mechanism is found to be much more efficient than a continuous notification of the recognized low-level contexts. In the particular case of not receiving any low-level context information for a given context category, the finalization of the current low-level context is simply informed.

### 2.2. High-Level Context Awareness

**Figure 3:**
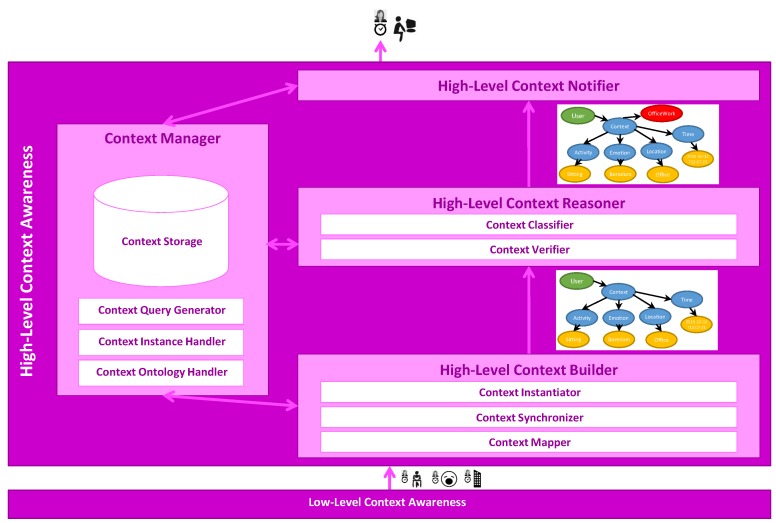
High-Level Context Awareness operation flow.

HLCA consists of four main components: High-Level Context Builder, High-Level Context Reasoner, High-Level Context Notifier and Context Manager. The operation of HLCA is as follows (Figure 3). The High-Level Context Builder receives unstructured low-level information, namely activities, emotions and locations, yielded by the Low-Level Context Architecture. Then, based on the received low-level context information, the High-Level Context Builder generates the ontological concepts representing the user high-level context. The unclassified high-level context is served to the High-Level Context Reasoner for its verification and classification into one of the different high-level context categories by applying ontological inference. Once a newly classified high-level context has been identified, the High-Level Context Notifier makes it available to any third party application that registered for this type of information. During the context identification process, several components interact with the Context Manager, which provides ontological persistence, also supporting the easy access to low-level context and high-level context information. For all of the aforementioned processes, HLCA uses the Mining Minds Context Ontology [21].

#### 2.2.1. High-Level Context Builder

The High-Level Context Builder receives the low-level information yielded by the Low-Level Context Architecture and generates the ontological concepts representing an unclassified high-level context associated with that information. The High-Level Context Builder has three subcomponents: the Context Mapper, the Context Synchronizer and the Context Instantiator. The Context Mapper interprets the received low-level information and transforms it into the corresponding ontological concepts. Once the low-level context instance has been created, it is stored in the Context Manager for its persistence, and this is notified to the Context Synchronizer. A change in the low-level context implies a new high-level context, comprising the new low-level context (e.g., a new detected activity) and the other low-level contexts (e.g., the emotion and location taking place), which are still valid at the start of the new low-level context. Therefore, the Context Synchronizer determines the other low-level contexts of a given user, which are valid a the start time of the new low-level context instance. In order to search for the concurrent low-level contexts, the Context Synchronizer requests information stored in the Context Manager and accesses it through the Context Instance Handler. The Context Instantiator is invoked when the Context Synchronizer has determined the low-level contexts concurrent to the one that triggered the inference process. The Context Instantiator creates a new instance of an unclassified high-level context, which links it to the comprising low-level contexts. Once the Context Instantiator has created the instance of an unclassified high-level context, this is served to the High-Level Context Reasoner for its verification and classification.

#### 2.2.2. High-Level Context Reasoner

The High-Level Context Reasoner performs a consistency check on the unclassified high-level context instance created by the High-Level Context Builder. In case the instance is valid, the High-Level Context Reasoner identifies the context type to which the high-level context belongs. The High-Level Context Reasoner comprises two subcomponents: the Context Verifier and the Context Classifier. The Context Verifier checks the semantic and syntactic consistency of the unclassified high-level context. Therefore, the instance of unclassified high-level context is validated and verified versus the Mining Minds Context Ontology. Once the Context Verifier has ensured that the unclassified high-level context is valid, this instance is provided to the Context Classifier. The Context Classifier identifies the type of high-level context to which the unclassified high-level context belongs; thus, converting the unclassified instance into a classified high-level context. The classification of the unclassified high-level context instance into one of the defined high-level context classes is based on the inference functionalities provided by the Mining Minds Context Ontology. Finally, the Context Classifier serves the classified high-level context to the High-Level Context Notifier.

#### 2.2.3. High-Level Context Notifier

The High-Level Context Notifier makes available to third parties the newly-identified high-level contexts. The High-Level Context Notifier receives from the High-Level Context Reasoner a classified high-level context instance and notifies the subscribed third parties about the detection of a new high-level context. As per the LLCA, this process is only conducted if the new instance belongs to a different high-level context type than the previous one. Likewise, only changes in the high-level context type are notified; this means that differences in the low-level context composition that do not imply a change on the type of high-level context are not communicated to the third parties. Furthermore, the High-Level Context Notifier stores the high-level context into the Context Manager for its persistence.

#### 2.2.4. Context Manager

The Context Manager persists the Mining Minds Context Ontology, including the terminology for the definition of context (i.e., the terms used for modelling both low and high-level contexts) and also the instances of context. Furthermore, it supports the easy interactions with the persisted information, facilitating the interactions with the storage infrastructure. The Context Manager has four subcomponents: the Context Storage, the Context Ontology Handler, the Context Instance Handler and the Context Query Generator. The Context Storage is a triple database store, which provides persistence for the storage of the Mining Minds Context Ontology. The Context Storage also provides read and write functionalities for the Mining Minds Context Ontology. However, this storage cannot be directly accessed, and all the interactions are handled through the Context Ontology Handler and the Context Instance Handler. The Context Ontology Handler provides the management functionalities to interact with the Mining Minds Context Ontology terminology stored in the Context Storage. This component enables loading the context ontology to the Context Storage at system start time. The Context Ontology Handler also supports the retrieval of the context ontology, so that the rest of components of HLCA have access to the latest version of the ontological terminology. Furthermore, the Context Ontology Handler enables the extension at runtime of the context ontology, for example including new types of low-level contexts and new definitions for the high-level contexts. Every time the ontology is updated, the other components of HLCA making use of the context ontology are notified in order to obtain an updated version of the terminology. The Context Instance Handler deals with the retrieval and storage of context information in the Context Storage. The Context Instance Handler offers three different functionalities: storage of a newly-mapped low-level context, retrieval of concurrent low-level contexts and storage of a newly-inferred high-level context. The Context Instance Handler poses to the Context Storage the queries created by the Context Query Generator in order to retrieve the persisted context information. The queries are automatically created based on some information derived from the context instance that the Context Instance Handler provides to the Context Query Generator. The Context Query Generator is capable of generating several different queries depending on the expected outcome required for each specific use case scenario.

## 3. Implementation

An initial implementation of the proposed framework is detailed in this section. First, the different context-aware models designed for both LLCA and HLCA are described. Then, the specific technologies and tools used for the development of these models, as well as the different functionalities supported by the Multimodal Context Mining framework are outlined.

### 3.1. Models

All three categories of low-level context recognizers have been considered in this version for implementation. For the activity recognition, both inertial-based and video-based approaches have been developed. The Inertial Activity Recognizer builds on the 3D acceleration and 3D rate of turn data collected from both smartphones and smartwatches for the identification of eight commonplace activities, namely *Eating*, *Running*, *Sitting*, *Standing*, *Walking*, *Stretching*, *Sweeping* and *Lying Down*. A non-overlapping sliding window of three seconds is used for the segmentation of the registered data streams [25], and time and frequency features, namely mean, zero-crossing rate, maximum, minimum, standard variation, quartile, range and cepstrum coefficients, are extracted for their discrimination potential [26]. A simple, yet robust k-nearest neighbour model [27] is used for the classification stage. The k-value for the KNN model is particularly set to three, as it has been proven to provide good results in some related works and for different settings [28,29,30]. It must be noted that while the placement of the smartwatch is inherently restricted to either wrist, the activity recognition model has been defined in a way so it is independent of the position or orientation of the smartphone, thus freeing users from the need of placing the device on a specific body location [31].

The Video Activity Recognizer operates on depth video data, and it is designed to identify all of the aforementioned activities, but *Walking* and *Running*, as these are seldom observed in the considered indoor scenarios. The depth video data are preferred to traditional RGB video, since it has been shown to be more resilient to ambient noise and illumination artifacts [32]. From the depth video, a 3D skeleton coordinates model comprising the user’s body joints is derived [33]. The designed model uses a 50% overlapping sliding window to partition the video stream into segments of three seconds [34]. Each segment is characterized by extracting a set of spatial and temporal features. For the spatial features, the Euclidean distance between joint and joint and the angle between joint-joint vector and horizontal axis are measured, while for the temporal features, the mean and standard deviation of each joint distance and angle are computed for every sliding window [35]. The classification process is carried out through a decision tree model, which has been demonstrated to work well in prior related works [36].

The recognition of emotions is carried out in this case by using the Audio Emotion Recognizer. The developed model builds on the audio data recorded through the smartphone’s microphone during call conversations [37], and it is devised to identify four key emotions, namely *Anger*, *Happiness*, *Neutral*, and *Sadness*. Non-speech reduction techniques are employed to differentiate among voice and silences. The signal stream is split into segments of three seconds size, which are further subject to a randomization for privacy-preserving purposes. Low-level descriptors and functionals frequently used in this domain, such as signal energy, pitch, statistical metrics and mel frequency cepstral coefficients [38], are extracted from each sequential segment. Support vector machines are considered for the classification process [39] based on an RBF kernel with automatically tuned (grid-search) hyper-parameters *γ* (= 0.01) and C (= 1).

The identification of the user location is approached through the Geopositioning Location Recognizer. This model builds on the smartphone’s GPS sensor to determine the person situation. Both longitude and latitude coordinates are registered in a timely manner and input to a geolocalization service engine, here Naver Maps [40]. Personal maps are previously constructed by collecting the users’ places of interest, so that the resolved location provides a unique and personalized description of the situation of each particular user. In this implementation, the following personal locations are considered for identification: *Home*, *Office*, *Restaurant*, *Gym* and *Mall*.

The fusion or unification process is only implemented for the activity case, as it is the only low-level context for which various recognizers have been implemented. The fusion process is conducted by using a weighted majority voting approach, which has been shown to be more efficient than regular majority voting [41,42]. The weights are empirically estimated based on the recognition capabilities or performance of each recognizer. As a result, a 0.5076 weight is estimated for the inertial activity recognizer and 0.4924 for the video activity recognizer, in other words, the decisions made by the model based on inertial data are subtly favoured above the ones made by the recognizer building on the video data when both are present. The fusion takes place every three seconds so as to match the recognition rate of both Inertial and Video Activity Recognizers.

The HLCA relies on the Mining Minds Context Ontology model. The Mining Minds Context Ontology is an OWL2 ontology [43] that comprehensively models high-level context based on low-level context information. In this case, five commonplace high-level contexts are particularly considered for their inference, these are *Inactivity*, *OfficeWork* , *Exercising*, *HavingMeal* and *Housework*. A thorough description of the Mining Minds Context Ontology also including how the considered high-level contexts are defined upon the different low-level contexts can be found in [21]. A Description Logic (DL) reasoner implementing an analytic tableau method [44] is used to carry out the inference process.

### 3.2. Technologies

Both LLCA and HLCA have been implemented in Java using available open source libraries. The different signal processing and machine learning steps used in each of the recognizers are implemented by using the Weka API (v3.8) [45]. All of the components of HLCA build on Apache Jena (v2.11.2) [46], a semantic web framework, which includes some APIs for handling RDF [47], OWL [43] and SPARQL [48]. In the implementation of the High-Level Context Reasoner, Pellet (v2.3.2) [49], an open source OWL DL reasoner for Java, has been utilized in combination with Jena to enable the ontological inference functionalities. Furthermore, in the Context Manager, the Jena Triple Store, namely TDB, has been used as the Context Storage for the persistence of the Mining Minds Context Ontology.

Although the Multimodal Context Mining system could run on a local machine, it has been here deployed over a cloud environment in order to support scalability and limitless computational power. Two virtual instances on the Microsoft Azure public cloud environment [50] have been used for LLCA and HLCA, respectively. The communication between these two modules is implemented by establishing service contracts, which communicate by means of RESTful web services [51]. The communication between the Multimodal Data Sources, here depth camera (Kinect v2 [52]), smartphone (Samsung Galaxy S5 [53]) and smartwatch (LG G Watch R [54]), and the LLCA is implemented through sockets due to their high-performance for real-time communications.

## 4. Evaluation and Discussion

### 4.1. Experimental Setup

The Multimodal Context Mining framework builds on two separate levels of abstraction for the identification of user context. Both low and high levels not only differ from a conceptual or semantic point of view, but also from the way their context-aware models are built. On the one hand, the definition and validation of the HLCA model, i.e., the ontology, is performed in a knowledge-driven fashion. On the other hand, most LLCA models require a training and validation process based on pre-existing data. For this data-driven learning process, a dataset is needed describing exemplary situations in which the contexts of interest take place. Although there exist several datasets for the training and validation of activity, emotion and location recognition models, to the best of our knowledge, none of them embrace all three categories as a whole. Accordingly, a novel open dataset, hereafter the multimodal context mining dataset, has been collected as part of this work for supporting multimodal context awareness.

**Figure 4:**
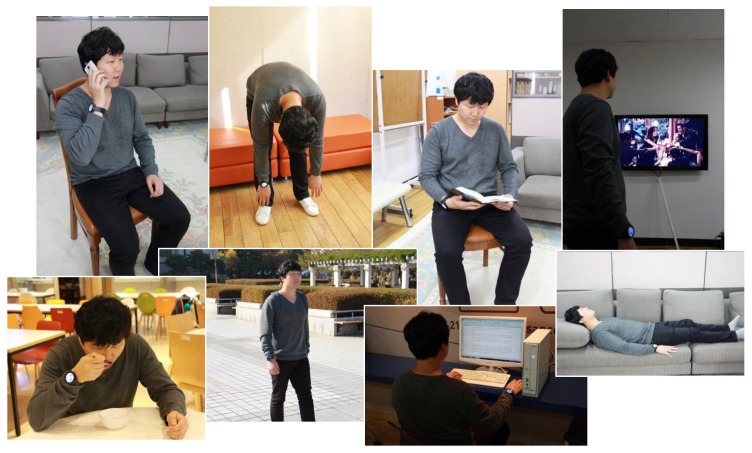
Examples of some of the low-level contexts collected as part of the multimodal context mining dataset.

**Figure 5:**
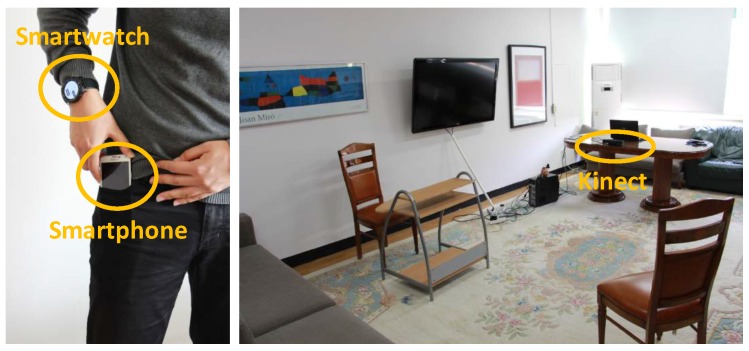
Sensor devices used for the collection of the multimodal context mining dataset. The smartwatch was generally placed by users on the right wrist, while the smartphone was kept in different locations based on the user’s choice. The Kinect video device was only used for monitoring in the home scenario.

The multimodal context mining dataset (available for download at http://www.miningminds.re.kr/technical-report/datasets/), also referred to as the Mining Minds dataset, comprises inertial, geopositioning, audio and video data collected for multiple users at various locations of Yongin city (Korea). A total of ten male subjects (S1–S10) were involved in the collection of the dataset (see Table 1). The volunteers were asked to perform a set of quotidian actions involving physical activities (*Eating*, *Running*, *Sitting*, *Standing*, *Walking*, *Stretching*, *Sweeping* and *Lying Down*), locations (*Home*, *Office*, *Restaurant*, *Gym* and *Mall*) and various emotions (*Anger*, *Happiness*, *Neutral* and *Sadness*). In order to procure a naturalistic collection of the contexts of interest, it was proposed to the users to freely execute similar related actions (Figure 4). Thus, for example, instead of explicitly requesting the users to sit down, the expert asked them to write a letter using the desktop computer. For the data collection process three devices were respectively used: a Samsung Galaxy S5 smartphone, an LG G Watch R smartwatch and a Kinect v2 video device (Figure 5). The smartphone and smartwatch were used for registering the user’s physical activity. The 3D acceleration and 3D rate of turn registered on the subject’s wrist (smartwatch) and another arbitrary body part to the user’s choice (smartphone) were collected at a 50-Hz sampling rate through the accelerometer and gyroscope sensors embedded into both mobile and wearable devices. Besides, the smartphone’s GPS sensor was used to register every second the longitude and latitude coordinates describing the location of the user. The smartphone was also used to record at 44.1 kHz the audio signals generated by the subject during some arbitrary phone calls intended to capture emotional states. For the home scenario, the depth video camera was used to also register the user’s body motion. The labelling of the dataset was carried out by using a remote application, which was handled by the expert or observer. All of the data collection sessions were also video taped to check anomalous or unexpected patterns in the data and to correct labelling mistakes during the posterior curation of the dataset. The entire dataset contains over ten hours of data, which accounts for approximately one hour per user in average. The duration of each of the considered low-level contexts is variable since subjects were not requested to perform any action for a specific period of time.

The evaluation of the multimodal context mining framework is carried out in two ways. First, an individual evaluation of the performance of each low-level context recognizer is conducted (Section 4.2). To that end, each model is cross-validated by using the appropriate data from the collected dataset. It must be noted that the high-level context inference cannot be strictly evaluated in an individual fashion since, unlike the low-level context recognizers, this model is not probabilistic, and as such, there is a deterministic relation between its inputs and outputs. In other words, the correctness of the inferred high-level context only depends on the correctness of the received low-level contexts. Second, a holistic evaluation is conducted in which the framework is assessed as a whole (Section 4.3). For this second case, the low-level context models trained on the collected dataset and the high-level context model are tested altogether at runtime for a group of independent subjects. Hence, this evaluation provides insights on the functional performance of each model during the regular use of the system and especially in relation to the inference of high-level context from low-level contexts.

### 4.2. Individual Evaluation

This section evaluates the performance of each of the implemented low-level context recognizers, i.e., Inertial Activity Recognizer, Video Activity Recognizer, Audio Emotion Recognizer and Geopositioning Location Recognizer. The multimodal context mining dataset (i.e., the data collected for S1–S10; see Table 1) is used for the evaluation process, which is here conducted through a leave-one-subject-out cross-validation process. The performance results are presented and discussed in the following.

**Figure 6:**
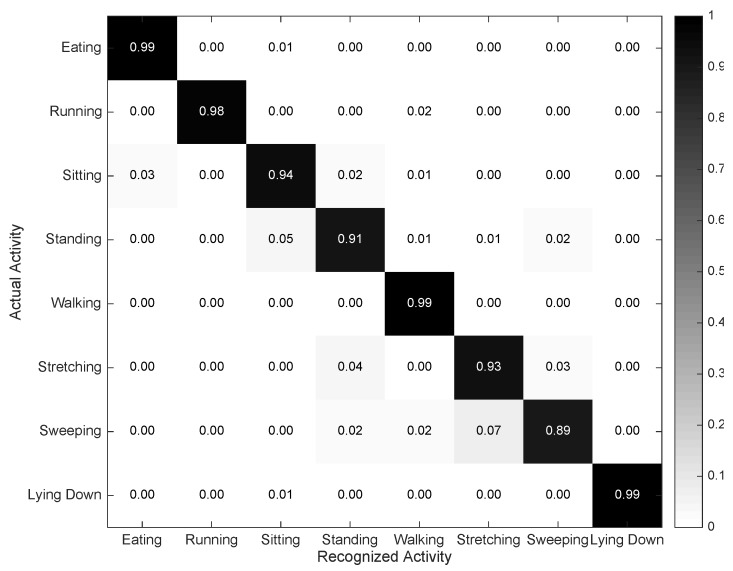
Confusion matrix describing the performance of the Inertial Activity Recognizer.

The confusion matrix for the Inertial Activity Recognizer is depicted in Figure 6. As can be observed, the developed recognition model seems to yield a high performance. After inspecting the confusion matrix, a few misclassifications can be identified for the pairs *Sitting*-*Standing*, *Sweeping*-*Stretching* and *Standing*-*Stretching*. Sitting and standing are passive activities involving low levels of motion; thus, the registered acceleration and rate of turn patterns tend to be more similar for these pairs of activities than for the others. Sweeping and stretching are sometimes misclassified, as both actions involve relatively similar movements from an intensity perspective. As a matter of fact, since users were given the liberty to execute all of the actions on their own, it was seen that some subjects bent their waist and stretched their arms notably while *Sweeping*, which matches with some of the observed stretching executions. This also applies for the *Standing* and *Stretching* case. On the one hand, some volunteers performed the stretching in a very light fashion, thus rather leading to low-intensity motions; on the other hand, various subjects moved the upper limbs while standing, movements that may coincide with some stretching patterns.

**Figure 7:**
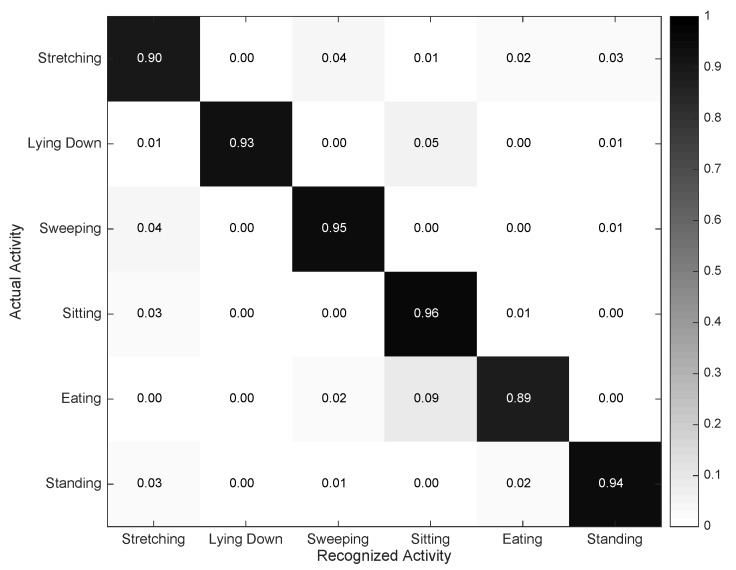
Confusion matrix describing the performance of the Video Activity Recognizer.

The performance results for the Video Activity Recognizer are presented in Figure 7. A lower set of activities are under the scope of this recognizer due to the restrictions posed by the home scenario, thus in theory leading to more accurate classification results. Yet, some misclassifications are observed. *Standing*, *Stretching* and *Sweeping* are in some cases misrecognized as per the reasons given for the inertial case. More prominently, *Eating* is sometimes recognized as *Sitting*. This is motivated by the fact that eating is performed while sitting, so strictly speaking, both activities take place concurrently or in an interleaved manner. Moreover, there are some resting periods in between intake actions, which could certainly be considered as sitting.

**Figure 8:**
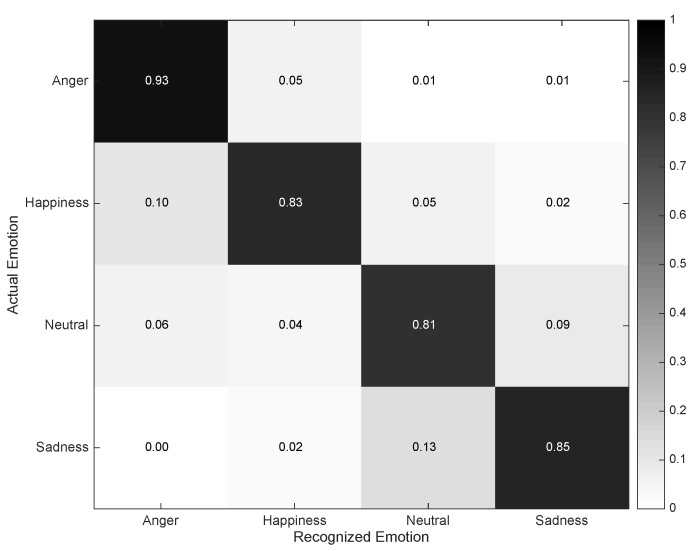
Confusion matrix describing the performance of the Audio Emotion Recognizer.

The confusion matrix for the Audio Emotion Recognizer is depicted in Figure 8. *Anger* and *Sadness* are most precisely identified, while worse results are obtained for *Happiness* and *Neutral* states. *Anger* and *Happiness* tend to be confused due to the loud tone used, for example while yelling or cheering. On the other hand, *Sadness* and *Neutral* are confused owing to the soft nature of the dialogues taking place during these states. Overall, the recognition capabilities shown by this model are more modest than for the previous recognizers. The reason for this rather mediocre result is fairly motivated by the complexity associated with registering realistic emotional states. While collecting realistic activity or location data turns out to be relatively feasible, even in the presence of experts or other people, reaching a realistic emotional performance is hard to attain in these very conditions. Apart from professional actors, people tend to feel embarrassed while conducting some fictitious dialogues involving certain emotions of interest. This is recognized to be one of the most important challenges in the emotion recognition and affective computing domains. More than that, other issues related to the large differences in tone, volume and pace among subjects make this task especially challenging.

**Figure 9:**
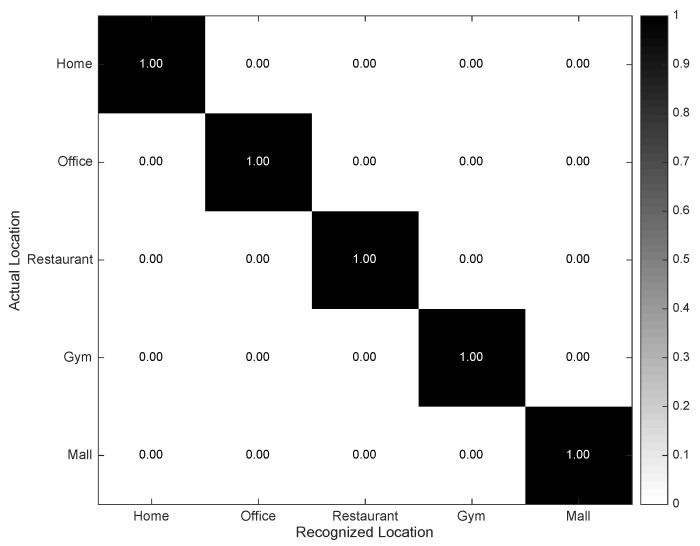
Confusion matrix describing the performance of the Geopositioning Location Recognizer.

Finally, the confusion matrix for the Geopositioning Location Recognizer is depicted in Figure 9. The results reflect a nearly absolute performance provided that a certain level of GPS signal is guaranteed. In fact, depending on the structure and dimensions of the building in which the user might enter or reside, there exist some chances of losing the GPS signal and, as a consequence, also the possibility of detecting the location. These situations were seldom observed in this experimental setup, possibly because of the robustness of the device and the characteristics of the considered environment.

### 4.3. Holistic Evaluation

**Figure 10:**
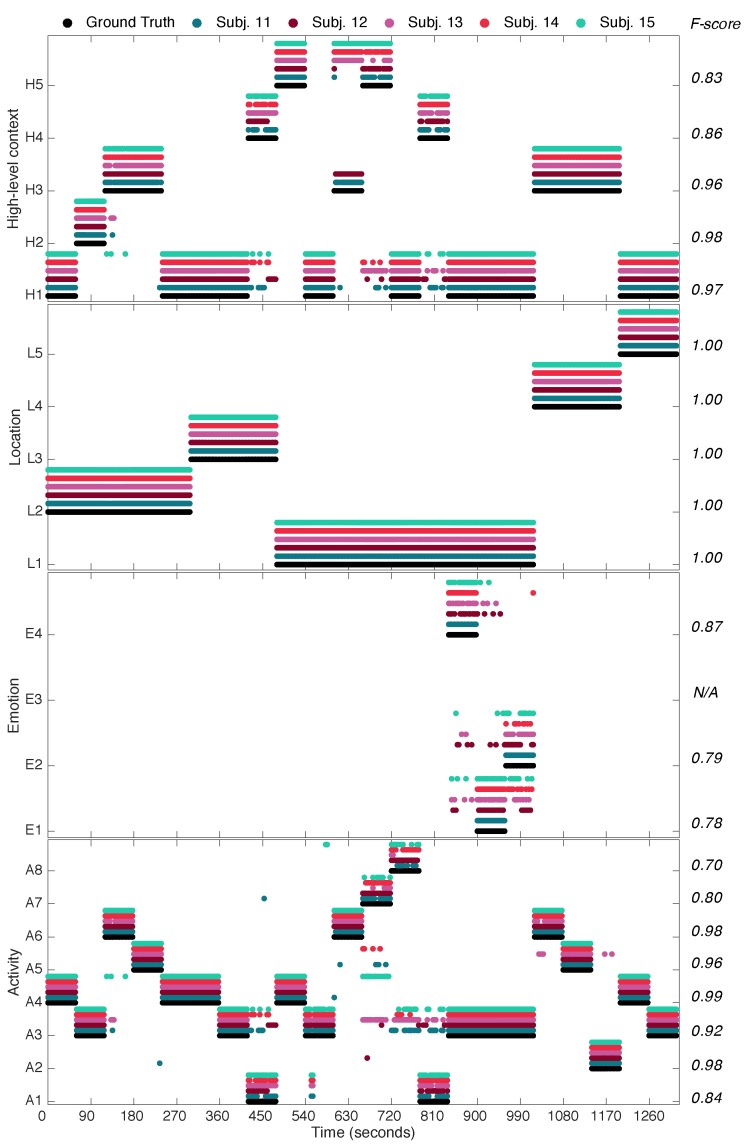
Low- and high-level contexts detected by the multimodal context mining system during online evaluation for the subjects S11–S15. Actual contexts are given by the ground-truth labels. Overall performance for each context and across all subjects is given by the corresponding F-score. Legend: A1 = *Eating*, A2 = *Running*, A3 = *Sitting*, A4 = *Standing*, A5 = *Walking*, A6 = *Stretching*, A7 = *Sweeping*, A8 = *Lying Down*); E1 = *Anger*, E2 = *Happiness*, E3 = *Neutral*, E4 = *Sadness*; L1 = *Home*, L2 = *Office*, L3 = *Restaurant*, L4 = *Gym*, L4 = *Mall*; H1 = *Inactivity*, H2 = *OfficeWork*, H3 = *Exercising*, H4 = *HavingMeal*, H5 = *Housework*.

An online holistic evaluation of the multimodal context mining system is conducted at this point to estimate the context recognition capabilities during realistic executions. The validation is performed on a different set of subjects to the one considered for the training of the models. A total of five independent volunteers (i.e., S11–S15, see Table 1) were asked to perform a run-through of actions involving most of the low and high-level contexts of interest. Each action was carried out during approximately one minute, and some of the contexts were executed various times during the run-through. The experiment took place in the same scenarios for which the multimodal context mining dataset was collected for the sake of simplicity and for the users’ convenience. A similar approach to the one used for the dataset labelling was used here for annotating the actual contexts. The contexts recognized during each run by the multimodal context mining system are contrasted against the registered ground truth, here depicted in Figure 10. Actual and predicted contexts are aligned in time taking into account the delay associated with the processing of the data (on average, 78 ms for the activity recognition, 77 ms for the emotion recognition, 0.46 ms for the location recognition and approximately 2 s for the high-level context inference). Transitions among the contexts of interest are left out of the study since a null-class rejection schema has not been explicitly implemented [55].

At an individual level, most of the findings already discussed in Section 4.2 for each type of context are further confirmed here. Thus, for example, activities such as *Walking*, *Standing* or *Sitting* are fairly recognized, while some misclassifications are observed for *Sweeping* and *Running*. More noticeable errors are nevertheless made while identifying *Lying down* and *Eating*, which tend to be categorized as *Sitting*. Some of these misrecognitions seem to be linked to the categorization of transitions of context, e.g., sit-to-stand or stand-to-sit, which is not supported by the current implemented models. For the emotion case, results are generally acceptable, yet there are a number of misrecognitions to be addressed. As discussed in the previous section, this is often the case due to the varying tone employed by users during the intended phone calls. The identification level is nevertheless absolute for the case of the locations, as it was already foreseen.

From a more global perspective, it can be further observed the impact that misrecognitions at the low-level have on the high-level inference process. It is clearly seen that a perfect identification of the high-level context is reached for those cases in which the recognized low-level contexts match the real ones. Examples of these cases are observed during the first hundred of seconds. Even in the event of low-level context misrecognitions, the performance of the high-level context remains nearly unaltered. This is for example observed during the executions taking place from the second 900 onwards. Around that time, various mistakes are encountered at the emotion level, which nevertheless do not drive to incorrect conclusions at the high level. This robustness is attained thanks to the way the high-level contexts are defined, for example by giving more importance to the performed activity or location than the emotional state. More prominent errors are observed around the second 450 and in the range 700–820 approximately. These erroneous inferences are as a result of the incorrect activities recognized at a lower level. Then, it can be concluded that there is in general a relevant dependency in terms of the performance for the high-level context on the low-level context.

Abrupt changes at the high-level context are rarely possible; thus, these fluctuations could be intelligently used to identify potential erroneous contexts at lower levels. In theory, by leveraging this idea, it could be possible to define a self-cleaning mechanism in which the detection of anomalous high-level contexts is used to debug, at runtime, potentially misrecognized low-level contexts, which could be then fed back to eventually correct the originally erroneous high-level context. It must also be noted that the nature of the individuals involved in this evaluation may pose some limitations on the generalization of the obtained results. As a matter of fact, the study focused on middle-aged male adults; thus, some variations can be expected in the performance of the recognizers of both activities and emotions when the models are trained with different data. No variations are nevertheless expected while detecting the user location since the positioning data are age and gender agnostic. This open issue presents a perfect opportunity to conduct a longitudinal study including a greater variety of age groups and both genders, which is here devised as part of the next steps of this work.

## 5. Conclusions

The analysis of people’s behavioural patterns and lifestyles is of much interest to prevent high prevalence diseases. Despite the significant progresses made to date, most existing solutions for determining these patterns only deal with one of the multiple facets of human behaviour. In light of this limitation, a novel approach for identifying human behaviour in a more holistic manner has been described in this paper. The proposed framework combines low-level context-awareness models devised to recognize activities, emotions and location primitives from multimodal person-centric data. This information is further considered to sophisticatedly infer more abstract representations of the user context, here identified as high-level context categories by using ontological reasoning techniques. An initial realization of the key architectural components has also been presented and evaluated in this work. The work is ongoing to complete the implementation of the devised architecture with new additional components, as well as to extend its evaluation to a large-scale testbed.

## Figures and Tables

**Table 1:** Characteristics of the participants involved in the multimodal context mining study. The height is given in cm, while the weight is measured in kg.

Subject	Age	Gender	Height	Weight
S1	29	Male	178	92
S2	27	Male	173	73
S3	28	Male	168	72
S4	27	Male	164	56
S5	24	Male	179	69
S6	25	Male	176	75
S7	25	Male	183	61
S8	22	Male	172	68
S9	24	Male	178	65
S10	30	Male	175	83
S11	31	Male	174	85
S12	25	Male	183	59
S13	29	Male	161	57
S14	27	Male	170	75
S15	30	Male	178	91
